# New York Pathological Society

**Published:** 1859-08

**Authors:** E. Lee Jones


### ART. V.—*New York Pathological Society.*

REGULAR MEETING, March 9, 1859. JOHN C. DALTON, JR., President.

(Reported for the Journal, by E. LEE JONES, M. D., Secretary.)

[Continued from page 104]

### VOLVULUS INTESTINORUM.

Dr. Henschell presented a specimen of volvulus intestinorum removed from a child 11 months old. The symptom during life was hæmorrhage from the bowels. The seat of the intus-susception was at the caput coli, the vermiform process and ileum being invaginated. There was no stegnosis of the rectum. He had seen a case somewhat similar to this, that Dr. Jacobi had.

### CYSTIC DEGENERATION.

Dr. Geo. T. Elliot presented a pair of kidneys which were affected with *cystic degeneration.* One weighed two pounds, the other ten ounces. They were removed from the body of a shoemaker, æt. 56, who was brought into the Hospital (Bellevue) in a moribund condition, suffering from apoplectic effusion upon the right side, there being paralysis of the left side of the body. No previous history could be obtained. He died very shortly after admission.

The brain presented the evidence of apoplectic effusion, the left lateral ventricle being distended by a clot which had broken through into the cerebral tissue in the neighborhood. The clot was about the size of a large English walnut. There was some fluid blood in the right ventricle. The two kidneys referred to were also found. The bladder was about half-full of urine, which was not examined.

He stated that the kidneys in themselves were not very rare specimens; but he had brought them chiefly with the hope that the exhibition of them might elicit from Dr. Clark an expression of his views upon the pathology of this scarcely understood affection; and some suggestions in regard to forming of a diagnosis.

Dr. Clark stated that inasmuch as Dr. Elliot had paid him the compliment of asking his views upon the subject, he felt bound to reply.

He remarked that it was well known that diagnosis in these cases was difficult, mainly because there were really no symptoms to indicate the presence of the disease until the last few days of life. These symptoms, said he, are often accompanied with suppression of the urine, and with evidences of cerebral compression. Under these circumstances, the physician is induced to notice the kidneys. Whether or not there was albumen in the urine in the early stage of the disease, he was unable to say ; he, however, suspected not.

In relation to the condition of the kidneys, he stated that he had had occasion to remark before to the Society, that his own conviction, derived from an examination of these diseased organs, was at variance with those expressed by others. There are three views, said he, advanced in regard to the cause of the changes that are noticed. One is, that the uriniferous tubes are closed at two different points by inflammatory action ; that the sacs become filled with the urinary secretion, which afterward becomes changed in its character; gradual accumulation of this fluid distends the sac so formed, to a greater or less extent, as the case may be. Another idea is, that the capsule of the Malpighian body is the seat of the disease, and becomes gradually dilated and dilated until it attains the size of these that are presented. The third conjecture was, that the individual cells of the epithelial lining of the tubes become greatly enlarged and vascular ; and that by their increase, these cysts were formed. He stated that if gentlemen would examine the kidneys, they would come to the same conclusion that he did a good many years ago, that neither of these explanations were applicable to these cases. He maintained that the cysts were a new production, that they were entirely outside the interlocular, in the midst of the blood-vessels. Each cell, said he, is complete of itself, and contains granular matter to a considerable extent, the smaller ones containing nuclei. Under the microscope, they were seen varying in size from something less than the diameter of the uriniferous tubes to eight or ten times their diameter ; increasing in size, they are capable, under some circumstances, of holding a pint of fluid. The kidney tissue, as these cysts enlarge themselves, is stretched between them, forming septa of healthy structures.

### TUMOR OF THE BRAIN.

Dr. Enos presented a specimen of *tumor of the brain* about the size of a hen's egg, removed from a lady sixty years of age. He saw her for the first time six weeks previous, in convulsions. He learned that she had one of these attacks last June, followed by numbness of the right side, with partial paralysis. From the time he first saw her, she continued to sink gradually from some disease of the brain, until she died, last evening. A short time before death, the paralysis was complete. Postmortem examination was made this afternoon. There was found the tumor alluded to, which was situated on the middle lobe of the left side. He stated that he had not examined the tumor under the microscope, but it looked very much as if it was cancerous in its character. The circumference of the tumor came to the periphery of the lobe, and in the immediate vicinity there were cysts containing fluid. The brain-substance in the neighborhood did not seem to be softened, though there was a considerable amount of redness present. She did not complain of any intense headache at any time.

Dr. Krakowitzer read the following history :

Caroline R———, 23 years of age, a native of Poland, of healthy parents; has never had any sickness except, living in a malaria place (city of Posen), intermittent fever five or six times, the last time when fifteen years old. Menstruated first at that age.

When seventeen years old contracted a severe disease, of what nature she is unable to say. She remembers that the most distressing symptoms were cough, copious expectoration, and shortness of breathing. Does not remember that there was any particular pain about the chest. She was confined to the bed for three months, and given up by her medical attendant. So, every medical treatment was discontinued, when she commenced recovering rapidly. Her menstruation did not return for six months; but in its place she had an attack of diarrhœa every four weeks, lasting two or three days. After her menstruation got regular, she was in the best imaginable health, without cough or short breathing, visiting entertainments and balls like every girl of her age, sometimes dancing a night through without any inconvenience. Whether all this is strictly true, it is difficult to say. A friend of the family present at the post-mortem examination, stated that she, once before being married, had had a child; of which fact her husband is entirely ignorant. Her mother, who was very circumstantial in her narrative of her daughter's life, never alluded to it.

She arrived in this country September, 1855, and continued in good health until her marriage, which took place in March, 1856. Her husband, who knew her for quite a time before he married her, never thought otherwise but that she was in perfect health, of florid complexion, fine, rotund form, of a gay and active temperament.

Soon after being married, she did not seem to go on so well, complaining sometimes of diminution of appetite and feebleness. She became pregnant in December, 1856. About that time she exposed herself, lightly dressed, to the protracted action of the cold air, and became immediately very sick with fever and great general *malaise*. The leading symptoms were want of appetite, great prostration, and swelling, which commenced at the ankles, ascending gradually to the hips. The size of the lower extremities is described as something enormous; certainly it was to such a degree as to make her unable to walk, even if she had not been confined to the bed from other causes. The face and upper part of the body kept free from swelling. Of her chest or the heart she did not complain; she coughed a little, and not often.

In June, 1857, when about seven months pregnant, without known cause labor set in, and she was delivered of a dead child. She did not have eclampsia; but neither did she improve any. Although under regular medical attendance from the commencement of her pregnancy, the nature of her disease seems not to have been suspected except by her third physician, ten weeks after delivery, who, for the first time, testing her urine by heat and nitric acid, found albumen in large quantity. Under his care she recovered very slowly, but it took nine to ten months after her delivery till she thought herself again in the possession of her former health. She enjoyed again good appetite and sleep, and gained once more her pristine fine complexion and fullness of form. The only thing that troubled her somewhat was palpitation of the heart, for the first time noticed as she recovered from her sickness, and getting easily out of breath, not so much in walking (she could promenade one or two miles without fatigue), but in going up stairs or attempting to assist in the household work. But being in pretty easy circumstances, she prudently abstained from anything which she knew would bring on palpitation of the heart. As an instance how well she could stand purely muscular fatigue, it is stated, that on the day of the great Atlantic-cable celebration (1st September, 1858), she was on her legs the whole day, till late at night; and that she bantered her husband in a joking manner, with being effeminate, when he complained next morning of feeling tired, saying herself that she was ready for the same exertion. In November, 1858, at a wedding she tried to dance, but had to desist before she got around the room, being quite out of breath. She got over this in a few minutes.

Soon after, the palpitation of the heart becoming troublesome, she consulted her last attending physician. She remembers that he had great difficulty in finding the pulse at her wrist, and did not always find it. In a couple of days afterwards, she became more sick, so as to be obliged to stay in bed. She was very feeble, short of breath, lost her appetite, and vomited frequently. These symptoms were most alarming about Christmas, 1858, when nothing would lie on her stomach, and it was thought that she might die any moment. Ice at last controlled the vomiting, and free use of moschus made her rally again. She recovered again a little, so as to have once more some appetite, and being able to go out a couple of times. Some time in the middle of January, 1859, she even went to the theatre; but reached home with the greatest difficulty, all the previous symptoms returning and continuing up to her death.

I saw her first February 14, 1859. Found her in bed, sitting, her body bent forward, resting her arms on a pillow before her, and supporting the drooping head with her hands. From this favorite posture she rarely parted, and only for short intervals, to rest the muscles, when they became tired, taking then a position on her side. The recumbent position, necessary for the examination of the anterior aspect of the chest and the abdomen, could only be maintained with the greatest inconvenience, increasing the already existing dyspnœa rapidly. The face was slightly tumid, with a languid, listless expression. The lips bluish pale. The skin of the whole body of a waxy paleness. The lower extremities only, and very little, œdematous. Conjunctiva and mucous membrane of the mouth pale, tongue somewhat coated. Respiration labored, rather short, 26 in a minute. Thorax well formed. Sound of percussion normal in front and behind, with the exception of a slight dullness from the angle of the scapula down—more so on the right side. Vesicular respiratory murmur everywhere, excepting from the angle of the right scapula down, where it was weaker. Sound of percussion dull and empty in the præcordial region, from the right margin of the sternum to a little beyond the papilla mammæ, and longitudinally from the third intercostal space to the epigastrium, passing without interruption into the dull and empty sound of the left lobe of the liver. No impulse of the *heart* visible, faintly to be detected by the touch in the fourth and fifth intercostal spaces, the mammary glands entirely shrunken, not offering any obstacle to palpation.

At the apex of the heart, instead of the first sound, a blowing murmur, growing fainter the more the stethoscope neared to the mesian line. The arterial sounds at the base of the heart, perfectly normal.

At the height of the second rib in the middle of the sternum, and extending somewhat to the left, instead of the first arterial sound, a blowing murmur, very similar to the one at the apex of the heart.

The liver protruding about two and a half inches below the margin of the ribs, its left lobe occupying the whole epigastrium, somewhat tender by pressure. No appetite, very little thirst. Vomiting almost every day, sometimes twice, of a watery, mucous substance, without bile. Number of contractions of the heart never more than ninety in a minute. Stools two or three times a day, watery, light-colored, without pain. Urine about a pint in twenty-four hours, of normal color, neutral. By testing with nitric acid, not quite so much by boiling, it formed a solid, whitish, lumpy mass which, allowed to settle, left about one third liquid urine above the coagulated albumen.

*No pulse*, not even at the brachial and femoral arteries. The exploration of the pulse of the carotids impossible, from the motions of the larynx, and more even from the inability to stand a continued pressure of the fingers at the side of the larynx.

*Diagnosis.*—Insufficiency of the mitral valve; no valvular disease of the aorta; atheromatous deposits, or small aneurism, at the arch of the aorta. Hydro-thorax in a moderate degree. Enlargement of the liver. Far advanced state of Bright's disease.

The want of the pulse in the large, accessible arteries was attributed to the extreme anæmia, and the feeble action of the heart.

From the fact that the commencement of Bright's disease coincided with the beginning of her pregnancy, whereas there were no symptoms of cardiac trouble except when she recovered slowly after her delivery, it appeared to me most probable that the kidney disease was the starting-point, and that the disease of heart was secondary, and produced by the kidney disease; a fact already noticed by Bright, and after him by many observers.

*Prognosis* very unfavorable.

*Treatment.*—Quinia and wine were given the first two days, but invariably thrown up. So, only pulv. Doveri, gr. v.—x., was given every night to secure some sleep.

The wretched condition of the patient never improved until she died, Feb. 26th, at 12 P. M., from exhaustion; symptoms of œdema pulmonum setting in two days before, and increasing up to death.

*P. S.*—Menstruation had ceased two months before death.

*Post-mortem, sixteen hours after death.*—Both *pleural sacs* containing clear serum, the right about two pounds, the left a little more than one pound.

Some old adhesions between the costal and pulmonal pleuras. The *lungs* throughout, very œdematous, but otherwise healthy.

In the *pericardium* a few drachms of clear serum.

*Heart* moderately distended. Longitudinal diameter from sulcus transversus to apex, 5″; transversal diameter at the julius transversus, 4″. Weight after removal of blood, with the pulmonary artery a little beyond its division, and with the aorta just past the origin of the left subclavian artery, but without the pericardium, ten ounces. All the cavities of the heart moderately filled with loosely coagulated, dark blood, with a few membranous deposits of white fibrin between some trabeculæ carneæ, mainly in the right ventricle. The atria, normal. The walls of the right ventricle $\frac{1}{4}''$ thick, its cavity considerably narrowed by the septum forming a strong convexity toward it. The conus arterosus and pulmonary artery, normal. Only *two* large valvulæ semilunares, but those normal and sufficient. The cavity of the left ventricle almost twice its normal size, the walls in an average $\frac{10}{16}''$, firm. The anterior curtain of the mitral valve, normal; the posterior one somewhat shortened and thickened at the free edge; the chordæ tendineæ of the first order, mostly matted to those of the second and third order, and inserting themselves on the free edge of the valve instead of its ventricular surface.

The origin of the aorta apparently closed by a rough calcareous mass occupying the point of insertion of the bases of the semilunar valves.

The arch of the aorta and its three branches, of normal size; its interior surface perfectly smooth and normal. On laying it open down to the valves, a similar calcareous obstruction presented itself to the eye. This mass had its seat exclusively in the exterior valve, mainly imbedded in its duplicature, thereby receiving an involucrum which covered it on the surface which looked towards the wall of the aorta; whereas, its inner aspect, looking towards the opposite valves, was bare from any covering, so that an irregular porous mass, resembling somewhat a coral in miniature, was in immediate contact with the blood. The anterior semilunar valve did not enter at all into the covering of the concretion, and the posterior one only with its exterior half. But this, as well as the whole anterior valve, was thickened and shrunken. So, for $\frac{3}{4}''$ the origin of the aorta was in one-half of its circumference constituted by a hard, porous, irregular concretion, losing apparently its character entirely, and leaving a sort of tortuous, irregular channel, just wide enough to allow a moderately-sized goose-quill to pass through.

The liver considerably enlarged, showing the familiar characteristics of nutmeg liver. The left lobe covering the stomach completely, which was contracted, its mucous membrane showing the usual changes of chronic catarrh. On the anterior margin of the right lobe of the liver, about $\frac{1}{2}''$ exteriorly from the gall bladder, there was a cyst imbedded in the substance of the liver, and protruding about three-fourths of its circumference out of it. A part of the omentum magnum was drawn up, and bound to the walls of the cyst by strong, short, old adhesions. The walls of the cyst were pretty thick, here and there interspersed with hard, calcareous layers of different sizes. Its contents consisted partly of grumous matter, partly of layers, looking like half-coagulated albumen, crowding each other in most variously shaped folds, somewhat after the fashion of the surface of condylomatous growths. As seen under the microscope, it consisted of detritus, oil-globules, a large number of cholesterin crystals, and the characteristic relics of the ecchinoccus, the hooks of its head, in any number.

The spleen twice its normal size, firm, its fibrous stroma richly developed.

Both kidneys much diminished in size, the right one weighing one and a half ounces, the left one not quite as much. Their bed of fat almost completely gone, the capsule very easy to be peeled off. The external surface irregular, nodulated. The more prominent noduli of a dirty, yellowish color; the substance between them of a dark, red, brown. On cutting open, the lines between the medullary and cortical substance almost entirely obliterated. The layer of cortical substance hardly thicker than $\frac{1}{8}''$, on some spots, the bases of the pyramids almost reaching to the surface of the kidney. Under the microscope, the epithelium of the Malpighian bodies, and of the Bellinian ducts, showing fatty degeneration. In many parts, no trace of glandular structure could be any more found, but only connecting tissue.

The *uterus* (not shown) of normal size and texture, a superficial erosion on the mucous membrane of the external os. The plexus pampiniformes very much distended with blood. In each ovary, a geometrically round blood-coagulum inclosed in a cyst, at some distance from the surface; the one in the right ovary of the size of a pistol ball, the one in the left the size of a small pea.

The *intestines* normal.

Dr. K. alludes to his error in diagnosis, as he had not suspected stenosis of the origin of the aorta. The absence of the pulse is very satisfactorily explained by the degeneration of the aortic valves. Certainly more weight ought to have been given to this symptom; but then the absence of any murmur at the root of the large vessels at the base of the heart excluded any probability of valvular disease of the aorta. If he was not mistaken in his examination, and he thinks not, as he found the arterial sounds at the base of the heart always clear and distinct,—he asks whether it is not possible, that with such an extreme stenosis of the aortic valves, and a very feeble action of the heart, no sounds at all might be produced, so that only the normal sounds of semilunar valves of the pulmonary artery could be heard? The blowing murmur, instead of the first sound, at the cartilage of the left rib, which seemed to him to indicate either aneurism or atheromatous roughness of the arch, was of course transmitted from the heart. Another interesting feature is the high degree of degeneration of the kidneys, being certainly of long standing, whereas its symptoms remained latent from spring, 1858, till the commencement of the winter of the same year.

Dr. Clark stated that in two instances, lately, of albuminous urine occurring in pregnant women, the sediment contained no casts; and the inquiry was made whether this was likely to be of so frequent occurrence as to be in any manner diagnostic. He thought it worth the inquiry, whether the fact was constant. In this case, it was his conviction that the disease of the kidneys began before pregnancy, and was the latent form that occasionally surprises us by a sort of explosion of characteristic symptoms.

Dr. Elliot had abundant opportunities for seeing all varieties of Bright's disease. He had seen women who became pregnant, pass on until near the close of their confinement, in good health, when they would be seized with convulsions. He had seen the urine of such women present the evidences of albumen, also containing blood corpuscles, casts of the uriniferous tubes, with healthy epithelial cells. He had seen those patients, after confinement, improve; that was to say, the urine became less highly charged with albumen, the casts became less numerous, the cells disappeared, until finally the urine became perfectly normal, and at the time they passed from under his observation, he had every reason to believe that all kidney trouble had subsided. He had tested the urine of 112 women under this circumstance, and all signs of albumen and trouble of the kidneys subsided in due time, with the exception of two cases.

He thought the researches of Dr. Isaacs threw a great deal of light upon disease of the kidney. He (Dr. I.) maintains that the Malphigian bodies do the greater part of the work. Dr. E. thought that the latency of the disease might depend in a great measure upon the integrity of those bodies.

Dr. Clark observed that the Malphigian bodies showed a great many changes; sometimes they were reduced to 1-4 their original size.

Dr. Ellict considered that those cases of kidney trouble have a good chance for recovery, where only albumen, epithelial cells, and blood-corpuscles are found in the urine; the prognosis was different, however, in those cases where fibrinous casts, oil-globules, albumen, and granular matter existed.

Dr. Peaslee remarked that there were two distinct forms of diseases of the kidney; one which occurred during pregnancy, and the other independent of it. There was a marked difference in the prognosis of the two, as well as in the microscopical characters. A woman may become pregnant, and by the pressure of the uterus upon the renal veins, cause congestion of the kidney, and albumen will make its appearance in the urine. This hyperemia of the kidney gradually subsides, as a general rule, and everything progresses favorably; but on the other hand, the disease may go still farther than this mere congestion, and granular degeneration may result, which will be permanent. He considered that the presence of casts was a grave affair. The other variety of disease of the kidney is fatty degeneration, which may exist during pregnancy, though it is not necessarily caused by it: this is the incurable variety.

Dr. Elliot thought that the physician should not be too hasty, in coming to a conclusion that no organic disease of the kidney existed when only albumen was present. If these cases were carefully watched, structural disease would in many cases show itself. The presence of albumen in many cases is attributed to certain articles of food, and the physician is very apt to look no farther, taking the effect as a sufficient explanation of the cause. In this connection, he referred to a student, who used to tell Christison, that he could bring on albuminuria at will, merely by eating cheese. This fact bothered Christison considerably, and he determined to watch the gentleman. So he did, and found ultimately that he died of Bright's disease.

Dr. Clark, although he did not have as much to do with the women as Dr. Elliot, recollects a case where albumen existed during pregnancy eighteen years ago, and the woman was now alive and well.

Dr. Elliot, in answer to a question from Dr. Markoe, stated that many of these cases have no œdema of the face. The patient may have convulsions of the most marked character. He recollected one case where such convulsions existed for two days, yet there was not the slightest trace of swelling of the hands, arms, or face, that would give rise to the suspicion of the existence of albuminuria.

### CYSTIC KIDNEY.

Dr. Clark presented a *cystic kidney*, removed from a cow, in Newark. It was brought to him by Dr. Lehlbac, of that city. The animal weighed, after having been dressed, 256 pounds; and it was regarded as very good beef. The kidney contained a large cyst about the size of an orange, in the centre of the organ, and another at one of the extremities. The whole organ weighed 15 ounces more than the average. The structure seems to have undergone fatty degeneration.

Dr. Clark next presented a specimen which he said was of more than usual interest to him, and he doubted not it would be to the Society. He gave the history as follows:

The gentleman from whom this heart was taken, was a patient of Dr. Van Kleek. He saw him four years ago for the first time. I then learned that he had been ill for three months; previous to which he had enjoyed as good health as other persons of his age, he being 65 years old. What he complained of this time was, that he was subject to attacks of insensibility, which would come upon him suddenly, and cause him to fall down. So frequent did these attacks become, that his friends would not allow him to go out without an attendant, for fear that he would injure himself by falling. At the time he saw him, he kept in the house most of the time, by way of precaution. He complained of a little disturbance of the heart's action. He, with the Dr., had an opportunity of seeing some of these peculiar attacks. He would be conversing, when suddenly his speech would cease in the middle of a sentence; in a moment his face would grow pale, his eyes be closed, and his chin drop upon his chest. He would remain in this position a few seconds, when he would gradually wake up, the color would slowly return to his cheeks, his eyes open; then his head would begin to move, and taking a long, full inspiration, he would assume the position in which he was before the attack, and take up the sentence that had been broken, and go on with it as if nothing had happened. These attacks occurred in such a way, and so frequently, that we were enabled to study the phenomena with some precision. Having found already some hypertrophy and valvular murmurs of the heart, he listened during one of these attacks; I would sit by the side of the patient, and with my ear to his chest, listen to his heart for 15 or 20 minutes at a time, while he was engaged in conversation. I would notice that the heart's action would become feeble; and would then raise my finger, and Dr. Van Kleek would notice that the fainting paroxysm would commence to show itself, and he would cease his talking; the heart would stop, and after an interval of little less than 15 seconds, it would commence to beat again. I would then raise my finger to the doctor, when he would observe that a little redness would come to the face, the head begin to move, and the patient would slowly wake up. We settled that point to our entire satisfaction.

This particular feature of his disease continued for 11 months, the symptoms of general disease of the heart not rapidly increasing. I saw him about a year before his death, three years after the first visit, with Dr. Van Kleek, when he was suffering from a very harrowing cough, considerable dyspnœa, and abundant expectoration. The murmurs were then substantially the same as at the first visit. The measurements of the heart at that time were as follows: From the median line along the left third rib, 4 inches; on the fourth rib, 5 inches; from median line to apex, 4½ inches,—making each of these measurements about an inch more than usual. The cough was so troublesome, and the expectorations so abundant, that it was difficult for his non-professional friends to believe that he had not consumption. This profuse bronchorrhœa continued, with more or less of dyspnœa till his death.

This latter event occurred in the following manner: About four days ago, he suddenly felt a sense of sinking and distress, and called to his wife who was by, to give him something, quick. She called the Dr., who was in the house at the time; but when he arrived at the chamber, he found the patient already unable to speak, with his pulse feeble, and sinking into a kind of collapse. In this state he remained for two hours, when he ceased to breathe.

### AUTOPSY.

At the post-mortem examination, this heart was obtained. We found that the pericardium was filled with dark-colored, bloody fluid, which we judged, through the walls of the membrane, to be blood. We took care to estimate what was the quantity of this blood, and in the next place where it came from. The fluid was removed by sponges, and was found to weigh ten ounces. The clot, which was found to equal the fluid blood in quantity, was not disturbed until the whole organ was removed. We may say, that there was from 20 to 22 ounces of blood in the pericardium. The next step was to find where it came from. There was no very great difficulty in determining that point. We found that there had been a rupture of the internal coat of the aorta, at a point nearly an inch above the aortic valve; and in the immediate neighborhood of this opening, were patches of atheromatous deposit. The rupture was about an inch and a half in extent in the circumference of the artery, splitting the layers of the middle coat, passing around the base of the aorta, penetrating all the coats, and escaping through the pericardium by a minute opening scarcely large enough to admit a small-sized probe. The opening was somewhat more than an inch below the original rupture. As regards the cause of the suspension of the heart's action, it is difficnlt to determine. The heart had undergone fatty degeneration, but not in an extreme degree. The mitral valve is the seat of calcareous deposit on its anterior and posterior margin; the aortic valves are also the seat of the same kind of degeneration.

Now, it does not appear after this examination, whether this interruption of the heart's action was due to an inelastic state of the aorta, to fatty degeneration of the organ, or some undiscovered change in the nerves supplying the organ. The termination of the case was purely an accidental one.

There is another interesting feature in the case, viz., a very extensive calcareous deposit between two folds of the pleura. This plate was about 5 inches in length, by 3½ to 4 inches in breadth. The two surfaces of the pleura in that situation were united, and seemed to have been so for a long time. There was no tubercular disease of the lungs. The attacks of syncope occurred in any position of the body—in the standing, sitting, or recumbent posture. I have seen a good many hearts the seat of fatty degeneration, but never before any one that presented this peculiar symptom.

### CANCER, INVOLVING KNEE JOINT.

*Dr. Voss* presented a knee-joint of a lady, 55 years of age, showing the existence of cancerous disease of the head of the tibia and fibula. About seven years ago, a tumor commenced to form on the lowest part of the knee, attended with shooting pains, and inability to use the joint. Her catamenia ceased when she was 50 years of age. The tumor gradually increased until it reached its present size. The prominent symptom was pain, which was so persistent and severe that amputation was advised, and accordingly performed. The deposit was mainly situated on the anterior aspect, and upon the articular surface, of the tibia, in such a manner as to make the tuberosity very prominent, and the concavity of the head of the tibia very marked. The deposit was distinctly cancerous. There was a tumor that showed itself on the posterior surface of the tibia. There was no external ulceration. The inner and upper part of the patella was eroded, which was also the case with the internal condyle of the femur. There was no hereditary predisposition; no affection of the lymphatic glands, or other organs, appreciable. Microscopical examination showed cancerous cells to exist in great quantity, with healthy bone-corpuscles.

---

Regular Meeting, March 23d, 1859.

Dr. Thomas M. Markoe presented a specimen of necrosed bone embracing a part of the tibia of a boy ten years of age, who had suffered a compound fracture of the leg. The laceration of the soft parts was such that a portion of the bone was exposed to a considerable extent; especially was this the case at the end of two or three weeks after the accident, when suppuration was fairly established. At the end of that time the bone was not only exposed, but in part dead. I dilated the opening, said he, and found that the bare bone was a fragment embracing the whole of the tibia at that point; part dead and part alive, I separated the periosteum, and removed this portion. The fibula was well in place. Properly adjusted splints prevented the fragments from drawing towards each other. The union of the fibula in due time became complete, and at the time he left the house, the dead portion of the tibia removed was entirely replaced by new bone; in short, his compound fracture was repaired as if no dead bone had been taken away. He stated that the specimen in its practical bearings was similar to those presented by Dr. Wood at the last meeting, and illustrated strongly the advantage of the mode of treatment employed. It illustrates, also, said he, that when necrosis of these fragments of bones takes place, it does not always implicate the whole fragment. That is, a fragment of this size may be exposed, and its anterior surface die, and while its central portions retain their vitality, its posterior surface may also die. The anterior portion may perhaps come away easily enough, but deep in the center of the callus will be a sequestrum from the back part of the bone which will be difficult to reach, and difficult to discover. That this is the case, sometimes, is well shown by the specimen in question. This circumstance of the death of the deeper laminæ of the separated fragments is one which is also brought to our notice in cases of necrosis from other causes.

He had seen an account of the regenerative power of the periosteum, in the Gazette Médicale, by M. Ollier. This gentleman shows not only that the periosteum is capable of regenerating bone, but when removed in a layer from the bone and pressed away from the other, tissues will produce in its new situation a layer of bone upon its surface. Still further, that when laminæ or shreds of periosteum are turned up in this way, sometimes in one shape and sometimes in another, whenever this periosteum traverses the soft parts it will produce bone upon its surface. Still further, he shows that by separating a piece of periosteum from the bone, and transplanting it in other parts of the body, this membrane would, in its new situation produce more or less bony deposit. It seems to me that these experiments are well worth our notice, and if reliable, are a positive addition to our knowledge in reference to the regenerative powers of the periosteum.

Dr. Clark stated that the experiments referred to were but the enlargements of the old experiments of Du Hamel. He placed silver tubes in the shafts of bones, and found that by the displacement of the periosteum, bone was developed from it in unusual places. He believed that the first great triumph that surgery has achieved in this matter, was by the hand of the late Surgeon Blandau, of Paris, something like fifteen years ago. He, finding the clavicle almost entirely decayed, was able to separate it from the periosteum, leaving that membrane in its place, and after a time found that a new clavicle had formed, not having precisely the same shape as the original, but sufficiently firm, however, to form a substantial support to the shoulder. As far as he knew, the second triumph in the same direction was in the operations performed by Jas. R. Wood, in Bellevue Hospital, on the lower jaw. The fact that bone can be reproduced from the periosteum is a new feature altogether, and is as Dr. Markoe remarks, strongly illustrative of the bone-preserving power of the periosteum.

Dr. Wood stated that the experiments of Du Hamel proved, that bone was generated from the periosteum not only laterally but longitudinally, from the ends of the fractured portions. It is this explanation of the formation of new bone which is so important to the surgeon in the treatment of fractures, and which has been illustrated by Dr. Markoe's case. I have had two cases where I have removed the whole shaft of the tibia from the head of the bone, down to the malleolus. Both these cases were condemned by consultation in the outset; they were both cases of Hospital gangrene, and so bad were they that the surgeons thought them unfit for amputation. Both these cases were placed by themselves, and by careful watching, and the gradual enucleation of the dead portions, they finally left the Hospital with new tibias, or at least, each with a tibia larger than the old one, and answering in every particular as good a purpose. The process was indeed a tedious one, but I was repaid more than twice over by the gratifying results. In almost all cases where you have constitution sufficient to make new bone, you will, by proper adjustment, be able to fill up the space left vacant by the removal of any fragment, by new bony tissue. The majority of these cases, until within the last few years, were amputated, because the appliances to bring about a proper result were not of the right kind. I remember very well before the bran splint was introduced into the N. Y. Hospital by Dr. Gurdon Buck, amputation in these cases were pretty uniformly resorted to. Dr. Buck showed what could be done by proper appliances, and demonstrated satisfactorily that these cases could be cured without resort to the knife; even compound fracture at the ankle joint where loose bone was present. In Bellevue Hospital we have been enabled by these means to save a number of limbs, where amputation was resorted to not longer than ten years ago. As I stated at the last meeting, conservative surgery is rapidly gaining the ascendency. If we can prevent deformity, and gain the utility of a limb, we deserve more credit than those who by their bold strokes with the knife, have received so much credit as operators. I have a patient now in the Hospital, who would, under other circumstances, have lost his limb, where the whole circumference of the femur is necrosed, and is partially separated by the process of enucleation. I will present the specimen at some future meeting of the Society. The process of removing it has been necessarily a gradual one, for the reason that we wish to prevent deformity, and laceration of the soft parts, and allow nature to take her own course, which would not be the case if the separation of the fragment is prematurely made. This is an important fact to take into consideration in the performance of this operation, to allow the new bone to be deposited in sufficient quantities, and to be of sufficient strength to allow the functions of the limb to be performed before the removal of the dead portion.

Dr. Voss presented a salivary calculus from Wharton's duct, removed from an old woman fifty years of age. This was the third calculus removed by him in that situation. The diagnosis was easy; in two of the cases he could touch the calculus by means of a probe introduced into the duct. He stated that such a condition of things was very frequently met with in the horse, and was ascribed to the presence of foreign substances in their food.

One of the calculi occurred in the substance of the submaxillary gland, producing fistula. The stone was extracted with a great deal of difficulty, and the fistula closed up. In this instance, the surface of the stone was very much roughened. He stated that it was a remarkable fact that calculi occurred so seldom, either in the duct of Steno or the parotid gland. There did not seem to be any appreciable obstruction of the saliva by the presence of these bodies in the ducts. Their composition was phosphate of magnesia and carb. lime.

